# Thoracoscopic surgical atrial fibrillation ablation in patients with an extremely enlarged left atrium

**DOI:** 10.1007/s10840-021-01056-1

**Published:** 2021-09-16

**Authors:** Jolien Neefs, Robin Wesselink, Nicoline W. E. van den Berg, Jonas S. S. G. de Jong, Femke R. Piersma, WimJan P. van Boven, Antoine H. G. Driessen, Joris R. de Groot

**Affiliations:** 1 Department of Cardiology, Heart Center, Amsterdam UMC, University of Amsterdam, Meibergdreef 9, 1105 AZ Amsterdam, The Netherlands; 2 Department of Cardiology, Onze Lieve Vrouwe Gasthuis, Amsterdam, The Netherlands; 3 Department of Cardiothoracic Surgery, Heart Center, Amsterdam UMC, University of Amsterdam, Amsterdam, The Netherlands

**Keywords:** Atrial tachycardia, Minimally invasive surgery, Pulmonary vein isolation, Atrial fibrosis

## Abstract

**Purpose:**

Efficacy of pulmonary vein isolation (PVI) for atrial fibrillation (AF) decreases as left atrial (LA) volume increases. However, surgical AF ablation with unknown efficacy is being performed in patients with a giant LA (GLA). We determined efficacy of thoracoscopic AF ablation in patients with compared to without a GLA.

**Methods:**

Patients underwent thoracoscopic PVI with additional left atrial ablations lines (in persistent AF) and were prospectively followed up. GLA was defined as LA volume index (LAVI) ≥ 50 ml/m^2^. Follow-up was performed with ECGs and 24-h Holters every 3 months. After a 3-month blanking period, all antiarrhythmic drugs were discontinued. The primary outcome was freedom of any atrial tachyarrhythmia ≥ 30 s during 2 years of follow-up.

**Results:**

At baseline, 68 (15.4%) patients had a GLA (LAVI: 56.7 [52.4–62.8] ml/m^2^), while 374 (84.6%) had a smaller LA (LAVI: 34.8 [29.2–41.3] ml/m^2^). GLA patients were older (61.9 ± 6.9 vs 59.4 ± 8.8 years, *p* = 0.02), more often diagnosed with persistent AF (76.5% vs 58.6%, *p* = 0.008). Sex was equally distributed (with approximately 25% females). GLA patients had more recurrences compared to non-GLA patients at 2-year follow-up (42.6% vs 57.2%, log rank *p* = 0.02). Freedom of AF was 69.0% in non-GLA paroxysmal AF patients compared to 43.8–49.3% in a combined group of GLA and/or persistent AF patients(log rank *p* < 0.001). Furthermore, freedom was 62.4% in non-GLA male patients, compared to 43.8–47.4 in a combined group of GLA and/or female sex(log rank *p* = 0.02).

**Conclusion:**

Thoracoscopic AF ablation is an effective therapy in a substantial part of GLA patients. Thoracoscopic AF ablation may serve as a last resort treatment option in these patients.

## Introduction

Patients with symptomatic atrial fibrillation (AF) refractory to antiarrhythmic drugs (AADs) have a guideline recommended indication for catheter or surgical ablation (Cox Maze or thoracoscopic) [1, 2]. These techniques effectively reduce the incidence and burden of AF [3, 4]. However, the efficacy of catheter ablation decreases, as the left atrial size (measured by left atrial volume index (LAVI)) increases [5]. Previous studies suggest a similar decrease of efficacy of surgical ablation with an enlarged left atrium (LA) [6–8].

A giant left atrium (GLA), an extremely enlarged LA, is associated with an increased propensity for AF as a consequence of structural atrial remodelling (i.e. atrial fibrosis through the deposition of extracellular matrix proteins, atrial dilatation) [7]. Increased atrial wall surface allows more re-entrant circuits when the wavelength of the arrhythmia remains either unchanged (refractory period and conduction velocity unaltered), or does not increase proportionally to the LA enlargement [7]. GLA may be a primary condition or a condition secondary to AF or secondary to AF itself, and is frequently accompanied with mitral valve regurgitation.

Since AF patients with an extremely enlarged LA are also commonly refractory to AADs, a more invasive treatment approach (i.e. ablation) may be indicated. However, given the negative correlation between increased LA volume and procedural success (or freedom of AF), physicians may dissuade an invasive ablation and accept AF, employing a rate control strategy to reduce symptoms. However, data on efficacy of surgical AF ablation in patients with a GLA are limited to patients undergoing the Cox Maze procedure [9, 10]. These studies suggest a similar efficacy in patients with a GLA compared to patients without a GLA. Of note, in these studies, AF ablation was often combined with LA size reduction.

The efficacy of thoracoscopic AF ablation without atrial size reduction in patients with an extremely enlarged LA is unknown. We hypothesize that thoracoscopic surgical AF ablation in GLA patients will have a reduced efficacy compared to patients with a normal or less enlarged atrium, but will result in freedom of AF in a substantial proportion of the patients. Simultaneously, the absence of AF following thoracoscopic ablation may positively influence the mitral valve regurgitation, if present.

Therefore, the current study primarily aims to assess efficacy and safety of standalone thoracoscopic surgical AF ablation in patients with and without an extremely enlarged LA (further defined as GLA) after 2 years of follow-up. Furthermore, risk factors for AF recurrence will be determined and mitral valve regurgitation will be objectified by echocardiography before and after surgery.

## Methods

This was a single-center, prospective cohort study, including consecutive patients who underwent thoracoscopic surgical AF ablation between 2008 and 2018 at our tertiary referral center*.* Detailed patient characteristics, including medical history, drug prescription, echocardiography and rhythm monitoring, were prospectively obtained. Patients were found suitable for thoracoscopic ablation when diagnosed with symptomatic, paroxysmal or persistent AF (including long-standing) documented on ECG, Holter or pacemaker electrogram at least once in the 12 months preceding presentation. Main referral indications included advanced AF, usually persistent AF with an enlarged LA, previously failed catheter ablation or patient preference [6]. Main exclusion criteria were prior catheter ablation in the preceding four months, NYHA class IV heart failure or a history of radiation therapy on the thorax. The study was approved by the local ethics committee. All patients provided written informed consent. The study complies with the Declaration of Helsinki.

### Thoracoscopic surgical AF ablation

The thoracoscopic ablation strategy was published previously [11]. In short, all patients were subjected to bilateral thoracoscopic pulmonary vein isolation (PVI) (≥ 6 RF applications to the PV antrum with the Atricure Isolator® Synergy™ bipolar RF ablation clamp). In persistent AF patients, additional left atrial ablation lines were created conforming to the Dallas lesion set, and included a superior and trigone line (Atricure Isolator™ Transpolar™ pen or Coolrail® Linear pen) [12–14]. Patients were cardioverted to sinus rhythm before testing conduction block.

In all patients, the PV and left atrial ablations lines were tested for bidirectional block with epicardial electrodes connected to an electrophysiologic system in the operating theatre, as described previously [11, 13, 14]. The left atrial appendage (LAA) was amputated using a stapler device. A subgroup of patients underwent additional ablation of the four major ganglion plexi (GP) and Marshall’s ligament on top of the above described ablation strategy [6]. These were patients who underwent thoracoscopic ablation before 2010 and who were randomised to GP ablation in the AFACT study [6]. Evoked vagal responses were tested before and after GP ablation in all patients by an electrophysiologist in the operation theatre [11]. GPs were localized based on anatomical landmarks as well as based on high frequency stimulation evoked response, and subsequently ablated.

### Definition of GLA

In the literature, GLA has previously been described been defined by two echocardiographic findings: a large LA depicted by M-mode ECHO with a diameter > 65 mm and a left ventricular posterobasal wall bent inward and lying between the dilated left atrial cavity and left ventricular cavity [9, 15]. For the current study, we defined GLA as a LAVI of 50 ml/m^2^, since a LAVI of > 40 ml/m^2^ is defined as severely enlarged [16]. LAVI has been found to be more accurate to determine left atrial volume, as it takes the 3D morphology of the LA into account [8]. We used the biplane method to measure the LA volume. With this method, LA volume is measured at the end-ventricular systole (maximum left atrial volume) and is measured in both the four and two chamber view. Importantly, the LA appendage is not included in the measurement. The LA volume conducted from this method is then indexed for body surface area.

### Follow-up and outcome

The primary outcome was the efficacy and safety of thoracoscopic surgical AF ablation after 2 years of follow-up in patients with and without a GLA. It was also assessed for LAVI > 55 ml/m^2^. Efficacy was defined as freedom of any atrial tachyarrhythmia (AF, atrial tachycardia or atrial flutter) documented on ECG or on 24-h Holter lasting > 30 s in patients who discontinued AADs, according to the definition in the HRS/EHRA/ECAS consensus document [1]. A blanking period of 3 months postoperative was established, during which atrial tachyarrhythmias were not considered a recurrence of AF. All patients were followed for 2 years with regular outpatients visits in our medical center, ECGs and 24-h Holter monitoring at 3, 6, 9, 12, 15, 18 and 24 months. Furthermore, patients were encouraged to obtain additional rhythm recordings when symptomatic and all recorded ECG and Holter data from referring hospitals were collected and included in the rhythm monitoring analysis.

After the blanking period, all AADs were discontinued, unless patients remained in AF. Restart of AADs was a secondary outcome. Anticoagulants were continued in all patients with a CHA_2_DS_2_-VASc score ≥ 1 (unless solely based on female gender), irrespective of the (presumed) absence of AF or the exclusion of the LAA, and according to current guidelines [2].

During follow-up, echocardiography was performed to assess ventricular function, LA and valve morphology and function, as part of standard of care. Mitral valve regurgitation measured by echocardiography was compared preoperatively and postoperatively after six months.

The safety of the procedure was assessed by the occurrence of procedural related serious adverse events within 30 days postoperative: death, reoperation or (prolongation of) hospital admission. All patients were treated with colchicine 0.5 mg once daily for 30 days in the postoperative period to prevent pericarditis. All adverse events occurring within 30 days postoperative were considered procedure related and adjudicated by a cardiothoracic surgeon.

### Statistical analysis

For the comparison of normally distributed, continuous variables, an unpaired sample *T*-test was used; results were expressed as means ± standard deviations (SD). In case of not normally distributed, continuous variables, the Mann–Whitney *U* test was used; results were expressed as median with interquartile range [IQR]. Categorical variables were expressed as frequencies with percentages and were compared by the Pearson *χ*^2^ test.

For the primary endpoint, event-free survival was plotted and estimated by Kaplan–Meier curves and compared using the log-rank test. This was also performed for subgroup analyses for AF type and sex. It was assumed that GLA altered the efficacy independently. Due to this perceived interaction, clinical parameters associated with efficacy were assessed by univariable and multivariable Cox regression models with stepwise backward selection (removal criterion *p*-value > 0.10) for patients with a GLA separately. The hazard ratios (HR) with corresponding 95% confidence intervals (95% CI) were calculated. Statistical analyses were performed using R version 3.3.2. for Windows (R Foundation for Statistical Computing, Vienna, Austria).

## Results

Thoracoscopic surgical AF ablation was planned in 498 patients between 2008 and 2017, of whom 442 (88.7%) patients had sufficient echocardiographic and follow-up data for inclusion. At baseline, GLA was diagnosed in 68 (15.4%) patients (median LAVI: 56.7 [52.4–62.8] ml/m^2^), while the control group of 374 (84.6%) patients had a smaller LA (median LAVI: 34.8 [29.2–41.3] ml/m^2^).

### Demographic characteristics

Baseline characteristics are described in Table 1. Patients with a GLA were older years than patients without a GLA (61.9 ± 6.9 vs 59.4 ± 8.8 years, *p* = 0.02). Sex was equally distributed, namely there were 19 (27.9%) females in the group with a GLA compared to 97 (25.9%) females in the control group, *p* = 0.84. Persistent AF was more prevalent in patients with a GLA *n* = 52 (76.5%), than in controls *n* = 219 (58.6%), *p* = 0.008. However, time since diagnosis of AF was similar for both groups: GLA was: 4.0 years [2.0.-6.3] compared 4.5 years [2.0.-8.0] in non-GLA patients, *p* = 0.60.

**Table 1:** Baseline characteristics for patients with an extremely enlarged LA (GLA) and with a smaller LA (non-GLA). *BMI* body mass index, *CRP* C-reactive protein, *eGFR* estimate glomerular filtration rate, *GLA* giant left atrium, *IQR* interquartile range, *LA* left atrium, *LV* left ventricle, *PCI* percutaneous coronary intervention, *SD* standard deviation

	GLA patients (*n* = 68)	Non-GLA patients (*n* = 374)	*P*-value
LA volume index, ml/m^2^ [IQR]	56.7 [52.4–62.8]	34.8 [29.2–41.3]	< 0.001
Female, *n* (%)	19 (27.9)	97 (25.9)	0.65
Age, years (± SD)	61.9 ± 6.9	59.4 ± 8.8	0.02
BMI, kg/m^2^ (± SD)	27.3 ± 3.3	27.4 ± 3.9	0.81
AF type:			0.008
Paroxysmal, *n* (%)	16 (23.5)	155 (41.4)	
Persistent, *n* (%)	52 (76.5)	219 (58.6)	
AF duration, years [IQR]	4.0 [2.0–6.3]	4.5 [2.0–8.0]	0.60
LV ejection fraction, % (± SD)	48.6 ± 11.1	52.7 ± 10.1	0.003
Previous catheter PVI, *n* (%)	7 (10.3)	78 (20.9)	0.39
Previous myocardial infarction, *n* (%)	7 (10.3)	17 (4.5)	0.11
Valvular disease, *n* (%)	12 (17.6)	33 (8.8)	0.06
Previous PCI, *n* (%)	6 (8.8)	28 (7.5)	0.90
Previous cardiac surgery, *n* (%)	2 (2.9)	3 (0.8)	0.36
CHA_2_DS_2_-VASc score, (± SD)	1.5 ± 1.4	1.6 ± 1.1	0.60
0, *n* (%)	12 (16.2)	104 (27.8)	0.18
1, *n* (%)	22 (32.4)	117 (31.3)	
≥ 2, *n* (%)	34 (50.0)	152 (40.6)	
Congestive heart failure, *n* (%)	13 (19.1)	25 (6.7)	0.003
Hypertension, *n* (%)	33 (48.5)	160 (42.8)	0.46
Systolic blood pressure, mmHg (± SD)	136.4 ± 18.9	133.3 ± 20.5	0.22
Diastolic blood pressure, mmHg (± SD)	86.8 ± 11.9	82.2 ± 12.8	0.007
Diabetes mellitus, *n* (%)	4 (5.9)	22 (5.9)	1.00
Vascular disease, *n* (%)	9 (13.2)	41 (11.0)	0.74
Age 65–74 years, *n* (%)	26 (38.2)	116 (31.0)	0.31
Age > 75 years, *n* (%)	0	10 (2.7)	0.35
Antiarrhythmic drugs:			
Class IA, *n* (%)	1 (1.5)	9 (2.4)	0.86
Class 1C, *n* (%)	20 (29.4)	125 (33.4)	0.21
Class II, *n* (%)	39 (57.4)	178 (47.6)	0.18
Class III, *n* (%)	22 (32.4)	164 (43.9)	0.08
Class IV, *n* (%)	9 (13.2)	46 (12.3i)	0.97
eGFR mL/min/1.73 m^2^ (± SD)	60.3 ± 4.3	60.0 ± 6.5	0.71
NT-proBNP, ng/L [IQR]	515 [232–992]	241 [103–560]	< 0.001
CRP [IQR]	1.8 [0.7–2.8]	1.5 [0.7–3.0]	0.95

Other co-morbidities were similarly distributed in both patient groups, except for congestive heart failure, which was more prevalent in patients with a GLA, *n* = 13 (19.1%), than in patients without a GLA, *n* = 25 (6.7%), *p* = 0.003. Left ventricle ejection fraction was 48.6 ± 11.1% in GLA patients compared to 52.7 ± 10.1% in non-GLA patients, *p* = 0.003. Consistently, a significantly higher NT-proBNP: 515 [232–992] ng/L was observed in patients with a GLA compared to NT-proBNP: 241 [105–560] ng/L, *p* < 0.001.

### Surgical details

Pulmonary vein isolation was performed in all patients and acute isolation was achieved in all patients*.* Left atrial lesions (Dallas lesion set) were more frequently performed in GLA patients than non-GLA patients, 51 (75.0%) versus 217 (58.5%, *p* = 0.03) respectively, consistent with more persistent AF in the former group. Furthermore, GP ablation was performed in 29 (42.6%) patients with a GLA and 212 (56.7%) patients without a GLA, *p* = 0.04. Hospital admission time was similar for patients with a GLA 5.8 ± 4.5 and without a GLA 6.3 ± 19.9 days, *p* = 0.82.

### Freedom of AF

**Fig. 1:**
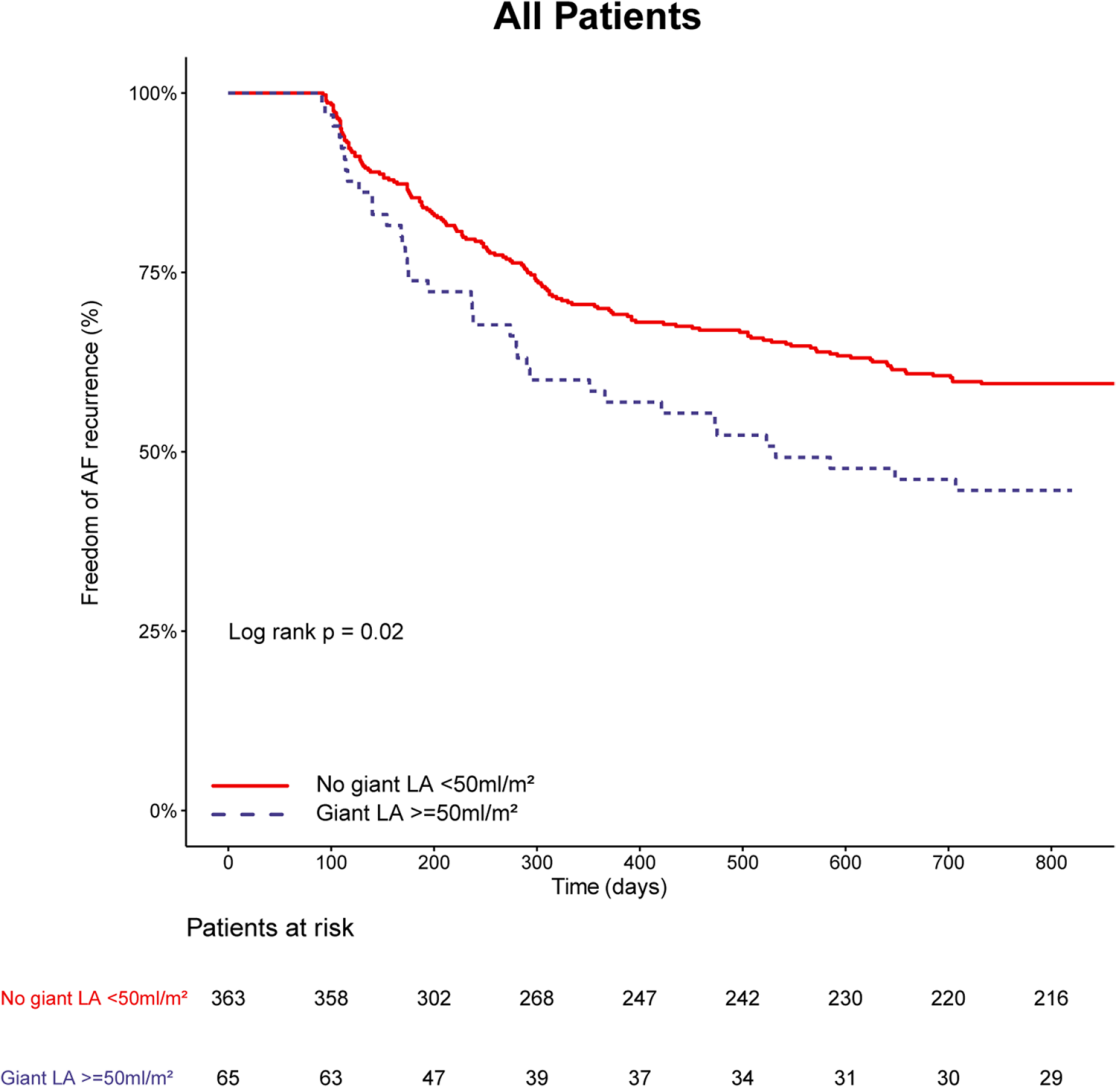
Kaplan–Meier analysis of AF recurrence in patients with an extremely enlarged LA (giant LA) and with a smaller LA (no giant LA). Giant LA: giant left atrium

At one year of follow-up, freedom of any atrial tachyarrhythmia lasting longer than 30 s was 55.8% (*n* = 38) in the GLA group and 67.9% (*n* = 254) non-GLA group (log rank *p* = 0.02). During the 2 years of follow-up, freedom of any atrial tachyarrhythmia lasting longer than 30 s was 42.6% (*n* = 29) in the GLA group and 57.2% (*n* = 214) in the non-GLA group (log rank *p* = 0.02) (Fig 1). After the initial blanking period, in these patients, AADs were thus not restarted. In the first year, AF recurred in 29 (42.6%) GLA patients, while another nine (13.2%) GLA patients had a recurrence in the second year. In comparison, AF recurred in 113 (30.2%) non-GLA patients in the first year, while 43 (11.5%) non-GLA patients had a recurrence in the second year. AADs were stopped after 3 months in all patients, but were restarted due to symptoms in 17 (25.0%) GLA patients compared to 76 (20.3%) non-GLA patients, *p* = 0.47. In the subgroup of patients with a normal LAVI (*n* = 177, mean LAVI = 28.7 ± 5.2 ml/m^2^), 117 (66.1%) patients were free of AF after two years of follow-up.

**Fig. 2:**
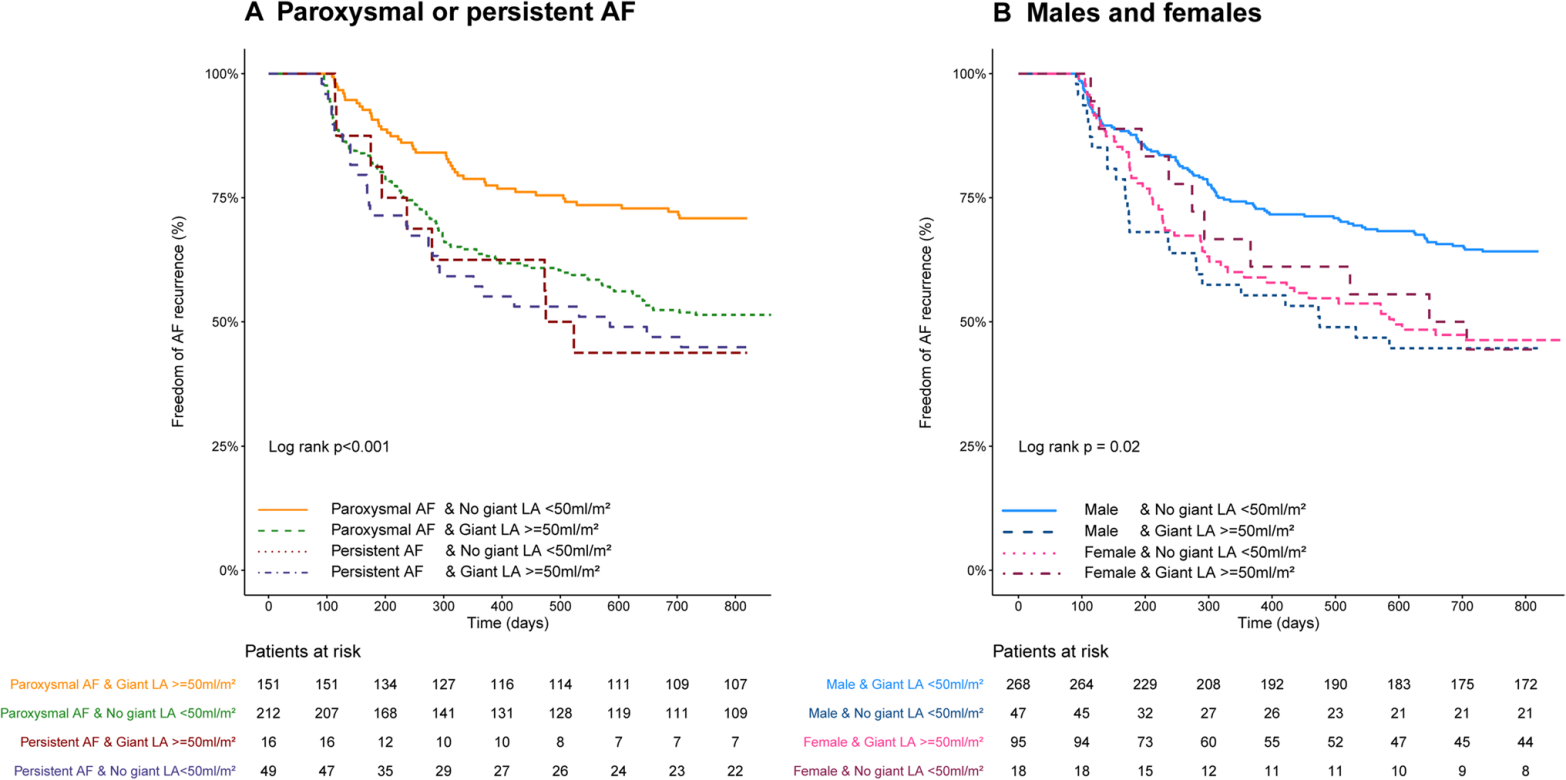
**A** Kaplan–Meier analysis of AF recurrence for AF type in patients with an extremely enlarged LA (giant LA) and with a smaller LA (no giant LA). **B** Kaplan–Meier analysis of AF recurrence in male and females in patients with an extremely enlarged LA (giant LA) and with a smaller LA (no giant LA). Giant LA: giant left atrium

Freedom of any atrial tachyarrhythmia was highest in paroxysmal AF patients (69.0%) without GLA at 2-year follow-up. Patients with either persistent AF, GLA or both, had similar freedom of AF, ranging from 43.8 to 49.3% for GLA and or persistent AF patients group (log rank *p* < 0.001) (Fig. 2A).

Furthermore, when stratified for sex, the highest efficacy of the thoracoscopic procedure was found in males without a GLA (success rate: 62.4%) compared to a significant lower outcome in males with a GLA, 43.8% at 2-year follow-up. Females had significantly lower freedom of AF than males without a regardless of the presence of GLA (43.8% with a GLA versus 47.4% without a GLA, log rank *p* = 0.02) (Fig. 2B).

In patients with a LAVI > 55 ml/m^2^ (*n* = 41, median LAVI: 61.2 [57.1–66.8]), freedom of any atrial tachyarrhythmia lasting longer than 30 s was 41.5% (*n* = 17).

### Type of atrial tachyarrhythmia recurrence

The type of recurrent atrial tachyarrhythmia tended to be differently distributed, *p* = 0.07. AF recurred in 17 (25.0%) GLA patients and 70 (18.7%) in non-GLA patients, while an atrial tachycardia was documented in 15 (22.0%) versus 71 (19.0%) patients, respectively. Atypical atrial flutter recurrence was the least common in both groups 5 (7.4%) and 10 (2.7%), respectively.

### Factors associated with freedom of AF in patients with a GLA

**Fig. 3:**
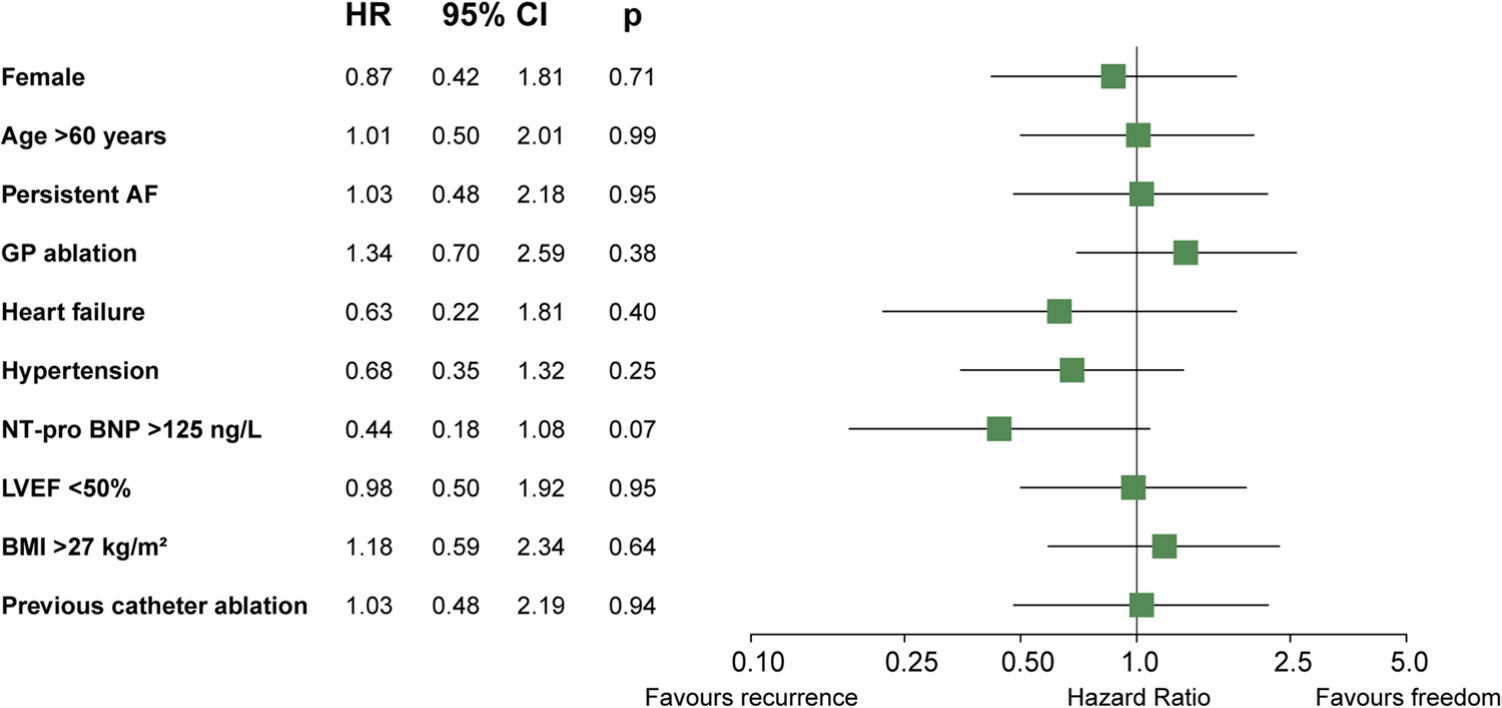
Forest plot of univariable and multivariable factors for freedom of AF in patients with GLA. 95% CI: 95% confidence interval; BMI: body mass index; GP ablation: ganglion plexus ablation; HR: hazard ratio; LVEF: left ventricle ejection fraction

Univariate analyses showed that an age under 50 years significantly favoured a successful outcome in patients with a GLA (HR: 5.30: 95% CI: 1.21–23.19, *p* = 0.03). No other clinical factors were significantly associated with efficacy. There was a trend towards a higher risk for recurrence for NT-proBNP > 125 ng/L (HR: 0.44: 95% CI: 0.18–1.08, *p* = 0.07). This was not seen in patients with a clinical diagnosis of heart failure or in patients with a left ventricle ejection fraction below 50%. After multivariable analysis, age under 50 years remained strongly associated with a successful outcome in patients with a GLA (HR: 5.84: 95% CI: 1.32–25.95, *p* = 0.02) (Fig. 3).

### Mitral valve regurgitation changes

Preoperative echocardiography showed that 33 (48.5%) patients with a GLA had mild, 6 (8.8%) patients moderate and 1 (1.5%) patient severe mitral valve regurgitation, while 100 (27.0%) patients without a GLA had mild, 20 (5.4%) patients moderate and 1 (0.3%) patient severe mitral valve regurgitation, *p* < 0.001.

**Fig. 4:**
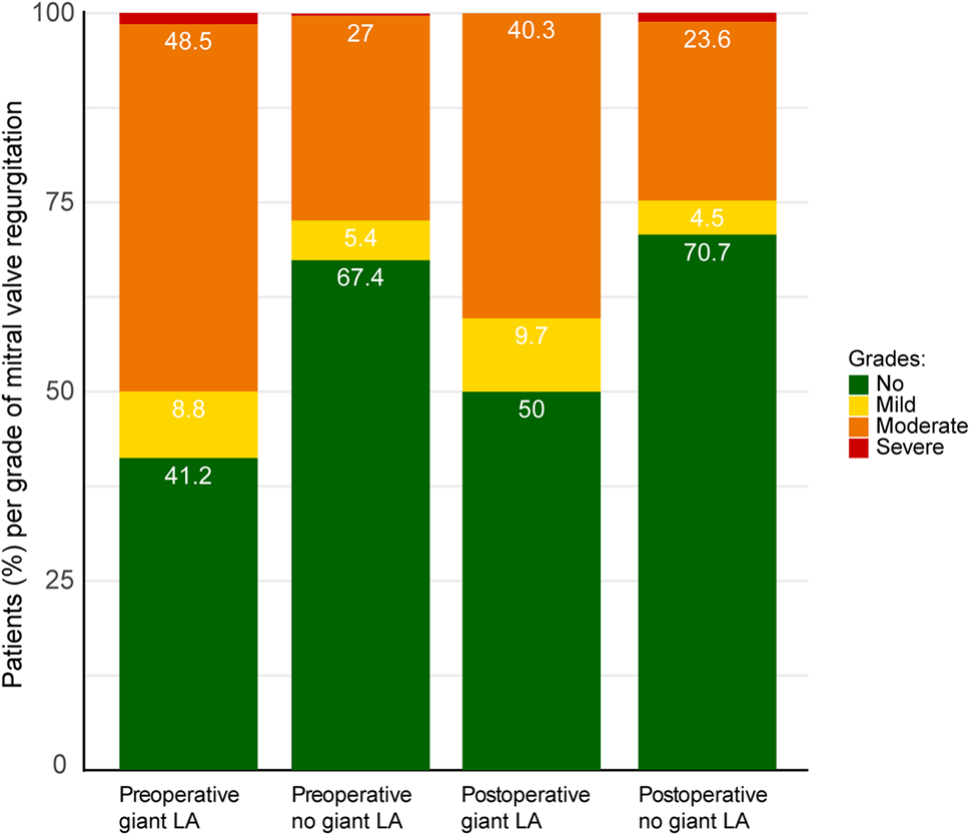
Ratio of grade of mitral valve regurgitation on echocardiography preoperative and postoperative for patients with an extremely enlarged LA (giant LA) and with a smaller LA (no giant LA). Giant LA: giant left atrium

During follow-up, echocardiography was performed in 434 patients, of whom 62 (14.3%) were patients with a GLA and 331 (76.3%) were patients without a GLA. Postoperatively, 25 (40.3%) patients with a GLA had mild, 6 (9.7%) patients moderate and 0 patients severe mitral valve regurgitation, whereas 78 (23.6%) patients without a GLA had mild, 15 (4.5%) patients moderate and 4 (1.2%) patients severe mitral valve regurgitation. There was a trend towards lower severity of mitral regurgitation after thoracoscopic AF ablation, irrespectively of the presence of GLA (Fig.  4).

### Adverse events

Procedural related serious adverse events within 30 days postoperative occurred in 68 (15.5%) patients (Table 2). No numerical difference in serious adverse events was found for patients with and without a GLA (data not shown). However, more pacemakers were implemented in the GLA cohort compared to non-GLA cohort (*n* = 3 (4.4%) versus *n* = 2 (0.5%), respectively, *p* = 0.03). In the total cohort, bleeding occurred in 26 (5.9%) patients, of whom 14 (3.2%) had a major bleeding that needed surgical intervention. In five (1.1%) patients, a sternotomy had to be performed to manage the bleeding, and seven (1.6%) patients had to undergo another thoracoscopic procedure. Furthermore, hemothorax was observed in seven (1.6%) patients. One (0.2%) stroke was observed and one (0.2%) pulmonary embolism. Lastly, phrenic nerve palsy was observed in three (0.7%) patients.

**Table 2:** Procedural related serious adverse events within 30 days after thoracoscopic surgery for AF ablation in the total cohort. *Indicated in case necessary to manage an adverse event, not to optimize the AF ablation

	Total cohort (*n* = 439)	GLA patients (*n* = 68)	Non-GLA patients (*n* = 374)	*p*-value
Death	0	0	0	
Stroke	1 (0.2%)	0	1 (0.3%)	
Reoperation*:				
Sternotomy	5 (1.1%)	0	5 (1.3%)	
Thoracoscopic surgery	7 (1.6%)	0	7 (1.9%)	
Bleeding				
Minor	12 (2.7%)	0	12 (3.2%)	
Major	14 (3.2%)	2 (2.9%)	12 (3.2%)	1.00
Hemothorax	7 (1.6%)	0	7 (1.9%)	
Pneumothorax (needing intervention)	11 (2.5%)	1 (1.4%)	10 (2.7%)	0.87
Pacemaker implantation (temporary or permanent)	5 (1.1%)	3 (4.4%)	2 (0.5%)	0.03
Phrenic nerve lesion	3 (0.7%)	0	3 (0.8%)	
Pneumonia	9 (2.1%)	0	9 (2.4%)	
Pericarditis	4 (0.9%)	1 (1.4%)	3 (0.8%)	1.00
Pulmonary embolism	1 (0.2%)	0	1 (0.3%)	

## Discussion

In this large cohort of patients undergoing thoracoscopic surgical AF ablation, we showed that 42.6% of patients with a GLA remain free from any atrial tachyarrhythmia during 2 years of follow-up. Freedom was similar in patients with a LAVI > 55 ml/m^2^. In comparison, freedom of AF was 57.2% at 2-year follow-up in patients with a significantly smaller LA. Furthermore, the low number of patients, in whom AADs were restarted, may also be considered a reflection of efficacy. Of note, we used the very stringent criteria of the HRS/EHRA/ECAS consensus document, in which any atrial arrhythmia recurrence lasting longer than 30 s is considered a procedural failure. These were patients with advanced forms of AF, in whom one or few short lasting recurrences of AF would confer an large improvement in their symptomatic arrhythmia burden. Indeed, we have previously shown that the quality of life after thoracoscopic ablation is the same for patients without AF recurrences and or one recurrence per year, whereas quality of life is lower in patients with more frequent recurrences [17].

A systematic review showed in patients with much smaller atria a significant higher success rate in patients who underwent surgical ablation compared to catheter ablation, but at the cost of more adverse events [3]. The patient category studied here comprehends patients with very enlarged atria unsuitable for catheter ablation due to diminished success rates. Next, the main indication for any invasive AF therapy is relieving symptoms. Driessen et al. [17] showed that despite recurrences approximately 85% of patients improved symptomatically after 5 years. Previous studies have reported very high success rates, but these results were derived from non-randomised studies and employing less strict follow-up [18, 19]*.*

Serious adverse events occurred infrequently and mostly comprised minor bleeding. Major bleedings could be controlled by thoracoscopic surgery in the majority of cases. These findings argue that thoracoscopic surgical AF ablation in patients with an extremely enlarged LA and symptomatic AF may be considered, but should be carefully weighted taken possible other significant cardiac conditions, patient preference and costs into account.

The highest efficacy of the procedure was observed in males with a relatively small LA, whereas in patients with an extremely dilated atrium efficacy of surgical ablation was similarly decreased for both sexes. A similar pattern was seen for AF type. Patient with paroxysmal AF without a GLA had the lowest risk of AF recurrence, in patients with persistent AF efficacy was similarly decreased for patients with and without a GLA. Successful outcome of the procedure was more frequently observed in young patients with a GLA. This is consistent with the findings of Lee et al. [10] in a cohort of patients undergoing the Cox Maze IIII surgery. No other clinical risk factors were associated with efficacy of thoracoscopic surgical AF ablation in patients with a GLA. The analysis may be limited due to the relative low number of GLA patients.

A considerable proportion of patients with a GLA suffered from heart failure, which may be caused by diminished LA contractility during AF, and aggravated by mitral regurgitation in patients with a dilated left atrium and ventricle. A GLA may require more energy to contract. From this, it has been suggested that a successful ablation relieves the burden of AF, improving contractility and through that way reduces heart failure [9].

### Mitral valve regurgitation

Echocardiography did not show a significant reduction in mitral valve regurgitation postoperatively, which may be explained by selection bias. In this cohort, most patients have no or mild mitral valve regurgitation and therefore no or marginal potential for improvement. Patients with more severe regurgitation do, mostly likely, not qualify for thoracoscopic surgery, but would opt for surgical mitral valve repair or replacement with concomitant AF ablation. Also, this cohort does not comprise patients with rheumatic mitral valve diseases, although rheumatic disease is a frequent finding in the general population of patients with a GLA [7]. In our cohort, a probable cause for LA dilation and secondary dysfunction of the mitral valve is the reduced quality of the atrial wall itself due to structure remodelling associated with AF. The structural remodelling of the atrial wall may further be negatively affected by the pressure overload as a result of mitral valve regurgitation [7].

### Atrial remodelling

An extremely enlarged LA is susceptible to AF due to structural and electrical remodelling (7,20). Vice versa, AF itself may cause enlargement of the LA. Structural remodelling of the LA through wall stress and chronic inflammation is associated with the deposition of atrial collagen, fibroblast proliferation and extra cellular matrix production leading to fibrosis [5, 7, 20]. Increased fibrosis will promote conduction slowing and repolarization heterogeneity and may further add to the vulnerability of AF recurrence.

Remodelling of the LA is more than only unidirectional dilatation. A previous study found that enlargement in the craniocaudal direction is more discriminative for thoracoscopic surgical AF ablation efficacy [21–23]. Moreover, deformations in the posterior atrial wall facilitate and maintain AF. Recently, it has been suggested that LAVI measured by echocardiography is limited by 2D and mathematical assumptions. Contrast-enhanced magnetic resonance angiography (CE-MRA) may be a more accurate option to assess dilatation and remodelling of the LA [8]. Specifically, patients with an extremely enlarged LA may benefit from careful assessment of size and morphology by CE-MRA for preprocedural planning. However, the definition used for this analysis makes it unlikely to have misclassified GLA.

### Safety of thoracoscopic surgical AF ablation

Patients with an extremely enlarged LA may be referred for ablation, since efficacy of catheter ablation is low and these patients may be more prone to complications [5, 24]. Consequently, in routine clinical care, a conservative medical approach may more often be adopted than in patients without a GLA. Importantly, the number of adverse events was not statistically different in both groups and comparable to literature [25]. The rate of major events (such as reoperation, pacemaker implantation and phrenic nerve lesions) were low, and in concordance with the complication rate in catheter ablation for AF [26]. The rate of pacemaker implantations reported in our study was lower than reported in the Cox Maze III or IV surgery [27]. Importantly, restoring sinus rhythm by surgical pulmonary vein isolation may also unmask pre-existing sinus node dysfunction for which a pacemaker is needed [28].

Previously, the AFACT study showed that GP ablation was associated with more adverse events [6]. The current analyses included patients who underwent additional GP ablation, which may have contributed to the number of adverse events. The significant higher number of patients with a pacemaker in the GLA cohort may indicate that these patients have more diseased atria, but importantly two patients of the three patients with a GLA and a pacemaker also underwent GP ablation. We have previously published that GP ablation is associated with a higher risk of pacemaker implantation after thoracoscopic AF ablation [6].

## Limitations

The current analyses defined GLA by as a LAVI ≥ 50 ml/m^2^, whereas Kawazoe et al. [15] also took LA deformation into account. LAVI has been shown to be a reliable measurement of LA volume, as it calculates enlargement using biplane images taking into account non-uniform enlargement [5, 8, 29]. The cut-off LAVI used to define GLA in this analysis resulted in an extremely high mean LAVI, making this group representative for patients with a GLA.

Possible selection bias may have occurred, since patients who underwent thoracoscopic AF ablation were relatively healthy, e.g. selection based on mitral valve function. Otherwise, it also shows that within patients with an extremely enlarged left atrium a considerable group of patients is in relative good condition and should be considered for surgical AF ablation.

Patients undergoing thoracoscopic AF treatment could not undergo surgical atrial size reduction, which may have affected the procedure’s success, but at the cost of a greatly more invasive and potentially more risky procedure. Of note, the amputation of the left atrial appendage in our cohort may have slightly reduced the left atrial size. Nevertheless, a substantial proportion of patients did not experience any atrial tachyarrhythmia during 2 years of follow-up and the majority of patients did not need to restart AADs.

We were not able to quantify AF burden, as no continuous rhythm monitoring was available. Regular 24 h rhythm monitoring with collection of ambulant ECG recordings ensures registration of nearly all symptomatic AF and provides systematic data on asymptomatic AF. More rigorous monitoring would inherently have resulted in a higher number of recurrences [1].

## Conclusion

The efficacy of thoracoscopic surgical AF ablation in patients with an extremely enlarged left atrium is substantial after two years of follow-up. In patients with an extremely dilated left atrium, sex and AF type do not affect efficacy of surgical ablation. However, younger age is associated with freedom of AF. Altogether, patients with an extremely enlarged LA may be offered thoracoscopic surgical AF ablation as a last resort option when symptomatic and without other significant cardiac conditions.

## Abbreviations

AF: Atrial fibrillation
GLA: Giant left atrium
LA: Left atrium
LAVI: Left atrial volume index
PVI: Pulmonary vein isolation
